# Content in YouTube Videos for Rosacea: Cross-sectional Study

**DOI:** 10.2196/24517

**Published:** 2021-02-10

**Authors:** Corey H Basch, Priscila E Laforet, Grace C Hillyer, Erica J Seidel, Christie Jaime

**Affiliations:** 1 Department of Public Health William Paterson University Wayne, NJ United States; 2 Division of Hematology/Oncology Department of Medicine Columbia University Irving Medical Center New York, NY United States; 3 Department of Epidemiology Mailman School of Public Health Columbia University New York, NY United States; 4 Department of Student Life/Counseling Center Borough of Manhattan Community College New York, NY United States

**Keywords:** rosacea, YouTube, social media, skin disease, skin, chronic, dermatology

## Abstract

**Background:**

Rosacea is an inflammatory skin disease that is chronic in nature. In addition to the physical symptoms, there are substantial quality of life issues that patients with rosacea experience, largely due to the visible nature in which rosacea manifests.

**Objective:**

The purpose of this study was to describe the content related to rosacea in highly viewed English- and Spanish-language videos on YouTube.

**Methods:**

We coded identifying information for each video and categories including characteristics of rosacea, clinical solutions, and alternative solutions. The 100 YouTube videos examined were viewed 18.5 million times between 2006 and 2020, and 57.3% (10,652,665/18,592,742) of these views were of consumer videos.

**Results:**

Videos posted by consumers more often promoted or were trying to sell a product or procedure (32/55, 58% of consumers vs 10/31, 32% of medical professionals and 4/14, 29% of television, internet, news, or entertainment sources; *P*=.03) and more frequently mentioned the use of makeup or other ways to cover up rosacea (30/55, 55% of consumers vs 6/31, 19% of medical professionals and 2/14, 14% of television, internet, news, or entertainment sources; *P*<.001). Videos sourced from medical professionals more often mentioned medication (17/31, 55%) than videos uploaded by consumers (14/55, 25%) or TV, internet, news, or entertainment sources (3/14, 21%) (*P*=.01).

**Conclusions:**

Given that rosacea is experienced differently for each person, consumer advice that works for one individual may not work for another. There is a need for reliable videos on rosacea to emphasize this and clarify misconceptions.

## Introduction

Rosacea is an inflammatory skin disease that is chronic in nature [1]. The cause of rosacea is unknown and the pathophysiology is inadequately comprehended [1]. Current statistics indicate that this is a prevalent problem, with an estimated 416 million adults affected [2] worldwide and an estimated 16 million individuals with rosacea in the United States [3]. Recent research has led to improved understanding of the common triggers and symptomology of this widely experienced issue. Triggers of rosacea include genetic and environmental factors [1], and patients are often encouraged to monitor environmental triggers closely to avoid the onset of symptoms.

The most commonly identifiable symptom of rosacea includes redness or flushing in the face, but the range of symptoms can be variable and are broken down into subtypes. The American Academy of Dermatology has identified 4 subtypes: (1) subtype 1, which is characterized by visible redness, flushing, and blood vessels; (2) subtype 2, in which acne-like breakouts are common; (3) subtype 3, which is rare and involves thickening skin that can result in rhinophyma, a thickened and bulbous nose; and (4) subtype 4, which affects the eyes with issues ranging from burning and stinging to the development of cysts [4]. It is possible to experience more than one subtype at a time. Naturally, because subtypes are varied and may overlap, treatments are dependent upon related symptoms and patient experiences [5]. These treatments include but are not limited to topical therapies [6-9], antibiotics [10], laser and pulsed light therapies [11-15], and reconstructive surgery [16,17].

In addition to the physical symptoms, there are substantial quality of life issues that patients with rosacea experience, largely due to the visible nature in which rosacea manifests [4]. Much has been written and researched about the medical aspects of rosacea, such as causes, prognosis, and treatment, but the psychological impact of the condition is infrequently discussed and of great importance to those with this condition. With limited ability to control triggers and the lack of a cure, patients are challenged with a lifelong chronic condition that alters their facial appearance, which often impacts their self-esteem and quality of life, especially when the rosacea is severe [18-21].

Current research suggests that 90% of Americans use the internet [22], and many consumers search the web for information related to their health. YouTube is a highly popular medium for sharing information through videos, with an estimated 2 billion unique users [23]. Studies of YouTube are prevalent on a variety of health issues and issues concerning the skin specifically [24-27]. The purpose of this study was to describe the content related to rosacea in highly viewed English- and Spanish-language videos on YouTube.

## Methods

The 100 videos with the most views on YouTube were identified using the keyword “rosacea” on May 31, 2020, and were recorded and coded. Videos in English and Spanish were included in the study. Six videos were not reviewed, as they were in a language other than English or Spanish, and they were replaced with the next 6 videos in English or Spanish.

Metadata were identified for each video, including the URL, source of video upload (consumer, medical professional, television- or internet-based news, or entertainment television), number of views, length of video in minutes and seconds, date of upload, language in which the video was recorded, and whether the video featured a medical doctor. A fact sheet from the American Academy of Dermatology was used to create coding categories for content [4]. Categories included characteristics of rosacea, clinical solutions, and alternative solutions.

Characteristics of rosacea included a general description of the condition, triggers and flare-ups of rosacea, the fact that rosacea is more common among women, and the impact of rosacea on the quality of life, such as feelings of frustration, embarrassment, worry, low self-esteem, anxiety, and depression. Mentions by subtype were noted. Specific details of the symptom subtype are noted below. Clinical solutions included mentions of surgery for thickened skin, laser treatment, medication, measures to protect against the sun, a potential cure, and the promotion of products or procedures. Skin care tips and mentions of makeup to cover the skin were included in the alternative solutions category. Responses were coded as “yes” or “no” for whether the video mentioned each of the above characteristics or solutions.

Frequencies and percentages of all categorical variables were calculated, and means, standard deviations, and ranges were determined for the number of views and video length. Video source was recoded as consumer, medical professional, or combined television-based news and entertainment television. Chi-square tests and analysis of variance were used to assess possible associations between video source and the characteristics and content of the videos. Authors EJS and CJ each coded half the videos and then coded a random 10% subset to ascertain interrater reliability. Using Cohen κ (κ=0.92), interrater reliability was shown to be excellent. Because human subjects were not involved in this study, this protocol was not reviewed by an institutional review board, per the policies at William Paterson University and Columbia University.

## Results

The 100 YouTube videos examined were viewed 18.5 million times between 2006 and 2020, and 57.3% (10,652,665/18,592,742) of these views were of consumer videos (Table 1).

**Table 1:** Characteristics of YouTube videos (N=100) about rosacea by video upload source.

Characteristic				Total (N=100)		Consumer (n=55)		Medical professional (n=31)		Television- or internet-based news and entertainment television (n=14)		*P* value
**Video characteristics**												
	Views, n (%)			18,592,742 (100)		10,652,665 (57.3)		5,527,057 (29.2)		2,513,020 (13.5)		N/A^a^
	Views, mean (SD)			185,927 (205,020)		193,685 (205,962)		175,066 (210,611)		179,501 (202,362)		.45
	Views, range			33,076-1,407,672		40,254-1,407,672		42,443-1,003,575		33,076-782,574		N/A
	Video length (min), mean (SD)			10.53 (8.92)		11.45 (8.60)		9.20 (7.53)		9.93 (12.60)		.42
	Video length (min), range			0.82-46.17		0.83-46.17		0.82-25.93		1.60-43.32		N/A
	**Video upload date, n (%)**											.15
		2006-2010	6 (6.0)		3 (5.5)		2 (6.5)		1 (7.1)			
		2011-2015	35 (35.0)		16 (29.1)		10 (32.3)		9 (64.3)			
		2016-2020	59 (59.0)		26 (47.3)		19 (61.3)		4 (28.6)			
	**Language of video, n (%)**											.03
		English	78 (78.0)		40 (72.7)		29 (93.5)		9 (64.3)			
		Spanish	22 (22.0)		15 (27.3)		2 (6.5)		5 (35.7)			
	Features a medical professional, n (%)			33 (33.0)		1 (1.8)		27 (87.1)		5 (35.7)		<.001
**Characteristics of rosacea, n (%)**												
	Included general description of rosacea			65 (65.0)		31 (56.4)		22 (71.0)		12 (85.7)		.09
	Mentions triggers and flare-ups			52 (52.0)		23 (41.8)		19 (61.3)		10 (71.4)		.07
	Mentions rosacea is more common in women			9 (9.0)		5 (9.1)		1 (3.2)		3 (21.4)		.14
	Mentions impact on quality of life			30 (30.0)		19 (34.5)		7 (22.6)		4 (28.6)		.51
	Mentions subtype 1 signs and symptoms^b^			65 (65.0)		31 (56.4)		22 (71.0)		12 (85.7)		.09
	Mentions subtype 2 signs and symptoms^c^			65 (65.0)		30 (54.5)		23 (74.2)		12 (85.7)		.04
	Mentions subtype 3 signs and symptoms^d^			33 (33.0)		12 (21.8)		14 (45.2)		7 (50.0)		.03
	Mentions subtype 4 signs and symptoms^e^			14 (14.0)		6 (10.9)		3 (9.7)		5 (35.7)		.04
**Clinical solutions, n (%)**												
	Mentions surgery for thickened skin			3 (3.0)		1 (1.8)		0 (0.0)		2 (14.3)		.03
	Mentions laser treatment			22 (22.0)		8 (14.5)		11 (35.5)		3 (21.4)		.08
	Mentions medication			34 (34.0)		14 (25.5)		17 (54.8)		3 (21.4)		.01
	Mentions sun protection			40 (40.0)		17 (30.9)		18 (58.1)		5 (35.7)		.05
	Mentions a cure			8 (8.0)		5 (9.1)		1 (3.2)		2 (14.3)		.41
	Promotes or sells a product or procedure			46 (46.0)		32 (58.2)		10 (32.3)		4 (28.6)		.03
**Alternative solutions, n (%)**												
	Promotes an alternative treatment			31 (31.0)		17 (30.9)		8 (25.8)		6 (42.9)		.52
	Mentions skin care tips			54 (54.0)		32 (58.2)		17 (54.8)		5 (35.7)		.32
	Mentions makeup or other ways to cover up rosacea			38 (38.0)		30 (54.5)		6 (19.4)		2 (14.3)		<.001

^a^N/A: not applicable.

^b^Subtype 1 signs and symptoms: flushing and redness, particularly in the center of the face; visible broken blood vessels and spider veins; skin that is swollen, very sensitive, or may sting and burn; rough, dry, or scaling skin; and skin that tends to flush or blush easily.

^c^Subtype 2 signs and symptoms: acne-like breakouts that tend to come and go and are found in the areas where the skin is very red, oily skin or skin that is very sensitive or may sting and burn, visible broken blood vessels and spider veins, and plaques with raised patches of skin.

^d^Subtype 3 signs and symptoms: bumpy skin or skin that begins to thicken, particularly on the nose, chin, forehead, cheeks, and ears; visible broken blood vessels and spider veins; oily skin; and large pores.

^e^Subtype 4 signs and symptoms: rosacea in the eyes where the eyes appear watery or bloodshot, feel gritty, burn or sting, itch, or are dry and sensitive to light; blurry or decreased vision; and visible broken blood vessels or a cyst on the eyelid.

The mean number of views was 185,927 (SD 205,020), and the mean length of the videos was 10.53 minutes (SD 8.92 minutes). Most videos were uploaded between 2016 and 2020 (59/100, 59.0%), recorded in English (78/100, 78.0%), and did not feature a medical professional (67/100, 67.0%).

Consumer videos less often mentioned signs and symptoms of subtypes 2 and 3 (subtype 2: 30/55, 55% of consumers vs 23/31, 74% of medical professionals and 12/14, 86% of television or internet; *P=*.04; subtype 3: 12/55, 22% of consumers vs 14/31, 45% of medical professionals and 7/14, 50% of television or internet; *P*=.03). Videos posted by consumers, however, more often promoted or were trying to sell a product or procedure (32/55, 58% of consumers vs 10/31, 32% of medical professionals and 4/14, 29% of television, internet, news, or entertainment; *P*=.03) and more frequently mentioned the use of makeup or other ways to cover up rosacea (30/55, 55% of consumers vs 6/31, 19% of medical professionals and 2/14, 14% of television, internet, news, or entertainment; *P*<.001). Videos sourced from medical professionals more often mentioned medication (17/31, 55%) than videos uploaded by consumers (14/55, 25%) or television, internet, news, or entertainment sources (3/14, 21%) (*P*=.01). Videos uploaded from a television, internet, news, or entertainment source more often mentioned subtype 4 (5/14, 36% vs 6/55, 11% of consumers and 3/31, 10% of medical professionals; *P*=.04) and surgical treatments for thickened skin (2/14, 14%) compared with consumer (1/55, 2%) and medical professional (0/0, 0%) videos (*P*=.03).

## Discussion

To our knowledge, this is the first study to examine the content of both English and Spanish rosacea videos on YouTube. The majority of the 100 most popular rosacea YouTube videos were uploaded by consumers. Thus, medical professionals should be aware that consumer opinions and thoughts on rosacea are accessed more often than professional materials. The type of information presented in the videos analyzed also varied depending on the source. Videos sourced from medical professionals were most likely to mention information on medication and the use of sun protection as treatments for rosacea, while videos sourced from consumers were most likely to mention information on alternative treatments like the use of makeup to cover up rosacea. Research indicates that cosmetics can exacerbate rosacea [28-30], and as such, the prevalent makeup tutorials related to covering rosacea could be promoting products that cause flares. In addition, rather than focusing on avoiding triggers, this content focused on hiding symptoms.

Within the context of the connection between self-esteem and body image [31], research is delving further into rosacea’s social and emotional fallout. Patients may avoid social situations, retreat from relationships, or think negatively about themselves as a result of their symptoms. An increase in symptoms of depression and anxiety related to the severity of the rosacea have been reported [19]. Women are more likely to be diagnosed with rosacea, exacerbating the gaps in self-esteem that already exist between men and women [32], suggesting that, for women, a holistic approach to treating the condition may be warranted to affect both the psychological and physical manifestations of the disease [33].

Further, the videos sourced from consumers were also found to be the most likely to include information to sell a product. This discovery highlights that consumers may have various underlying motivations to upload videos on rosacea, such as commercial sponsorship, which might result in the communication of misinformation to increase sales of a sponsored product to treat rosacea. Analysis of the videos revealed that the accuracy and reliability of the information found in the videos varied greatly. This is best highlighted by the videos that included information on a cure despite the fact that there is no cure for rosacea. The findings of this study are similar to a prior study of rosacea on a variety of internet sources, including YouTube videos, which concluded that internet sources could contain peer-generated content that was harmful or misleading [34].

This study has limitations that warrant mention. The cross-sectional design indicates that data were only collected at one point in time, and given the fact that content on the internet is in flux, the most popular videos could change over time. Additionally, this study only included videos in English and Spanish despite videos being available in an array of languages. Further, there is no way to delineate who viewed each video and the reason they did so. Therefore, the study strictly offers insight on the content and coverage of information in the widely viewed videos on YouTube.

Nonetheless, this study offers insight into the content available on YouTube about rosacea. Given that rosacea is experienced differently for each person, consumer advice that works for one individual may not work for another. There is a need for reliable videos on rosacea to emphasize this and clarify misconceptions. Further study is needed on the accuracy and reliability of information on rosacea in videos sourced by consumers, as well as on the factors that influence consumers to create and upload videos on rosacea for YouTube.
